# Synthesis and neuroprotective effects of the complex nanoparticles of iron and sapogenin isolated from the defatted seeds of *Camellia oleifera*

**DOI:** 10.1080/13880209.2016.1245346

**Published:** 2016-12-09

**Authors:** Qian Yang, Chuang Zhao, Jun Zhao, Yong Ye

**Affiliations:** School of Chemistry and Chemical Engineering, South China University of Technology, Guangzhou, PR China

**Keywords:** Nanomedicine, metallic chelate, structural analysis, utilization waste products, neuroprotection

## Abstract

**Context:** The defatted seeds of *Camellia oleifera* var. *monosperma* Hung T. Chang (Theaceae) are currently discarded without effective utilization. However, sapogenin has been isolated and shows antioxidative, anti-inflammatory and analgesic activities suggestive of its neuroprotective function.

**Objective**: In order to improve the activities of sapogenin, the nanoparticles of iron–sapogenin have been synthesized, and the neuroprotective effects are evaluated.

**Materials and methods:** Structural characters of the nanoparticles were analyzed, and the antioxidant effect was assessed by DPPH method, and the neuroprotective effect was evaluated by rotenone-induced neurodegeneration in Kunming mice injected subcutaneously into the back of neck with rotenone (50 mg/kg/day) for 6 weeks and then treated by tail intravenous injection with the iron–sapogenin at the dose of 25, 50 and 100 mg/kg for 7 days. Mice behaviour and neurotransmitters were tested.

**Results:** The product had an average size of 162 nm with spherical shape, and scavenged more than 90% DPPH radicals at 0.8 mg/mL concentration. It decreased behavioural disorder and malondialdehyde content in mice brain, and increased superoxide dismutase activity, tyrosine hydroxylase expression, dopamine and acetylcholine levels in brain in dose dependence, and their maximum changes were respectively up to 60.83%, 25.17%, 22.13%, 105.26%, 42.17% and 22.89% as compared to vehicle group. Iron–sapogenin nanoparticle shows significantly better effects than the sapogenin.

**Discussion and conclusion:** Iron–sapogenin alleviates neurodegeneration of mice injured by neurotoxicity of rotenone, it is a superior candidate of drugs for neuroprotection.

## Introduction

Neurodegeneration is a late-onset, progressive cognition and motor disease marked by neuronal loss or death with uncertain mechanism (Jabir et al. 2014). Parkinson disease (PD) and Alzheimer’s disease (AD) are typical in neurodegeneration with pathological hallmarks of α-synuclein and β-amyloid in neurons of cerebral cortex, striatum, thalamus, hypothalamus, amygdale and basal forebrain (Bae et al. 2012; Balducci & Forloni 2014). A majority of studies explored the effect of oxidative stress that contributes to the cascade of events leading to neurodegeneration (Venkateshappa et al. 2012). Currently, there is no perfect treatment for those diseases, and traditional treatment strategy is L-dopa replacement therapy for PD (Fernandez 2015), and acetylcholinesterase inhibitor for AD (Kumar & Singh 2015). Although the clinical symptoms are mitigated, disease progression cannot be stopped (Ohno et al. 2015). Moreover, long-term chemical treatment leads to efficacy reduction and severe adverse effects, lowering the quality of patients’ lives (Duty 2010). In recent years, medication therapy has changed from direct chemical treatment to multilevel therapy, aiming at reliving the symptoms and impeding the progression of the disease at the same time (Venigalla et al. 2015). Natural medicines play an important role in prevention and therapy of neurodegeneration (Zhao 2009).

*Camellia oleifera* var. *monosperma* Hung T. Chang (Theaceae), an edible oil plant grown only in the mountain region of China inland and Taiwan, differs from tea plant (Wei et al. 2012). The defatted seeds of *C. oleifera* are usually discarded leading to great waste of natural resource and environmental pollution. Recent research discloses that the defatted seeds of *C. oleifera* contain many bioactive compounds such as saponins, flavonoids, polysaccharides, etc. (Luo et al. 2003). Saponin is one of the active ingredients in many herbs, and natural saponins have neuroprotective effects relevant to the suppression of oxidative stress, elimination of free radicals and inhibition of inflammation (Han et al. 2008; Wang et al. 2010; Ye et al. 2011). Our previous researches proved the antioxidative, anti-inflammatory and analgesic activities of the sapogenin isolated from the seeds of *C. oleifera*, disclosing its central roles (Ye et al. 2013a, 2013b, 2014), and will further evaluate its neuroprotective effect in this research.

Iron, an essential element for growth and breeding, is the component of haemoglobin and many enzymes such as oxidase, reductase, etc., which consist of important antioxidant system *in vivo* (Valerio 2007). Iron takes part in many brain physiological process and formation of nerve myelin sheath and lots of neurotransmitters (Lozoff et al. 2006). Organic iron have greater bioavailability than the inorganic (Ma et al. 2014), but accumulation of inorganic iron in brain is harmful and relevant to neurodegeneration (Bellingham et al. 2015).

We found that the sapogenin could make a stable complex with iron, and synthesized an organic iron compound (iron–sapogenin) with nanoparticle characters, which inspired us to investigate its central effects. In this article, we evaluate the antioxidant effect of iron–sapogenin nanoparticle *in vitro* and *in vivo*, and investigate its effect on neurotransmitters in central tissues in order to assess potential as a new drug for neuroprotection.

## Materials and methods

### Chemicals and reagents

Sapogenin (purity 98%) was isolated from the defatted seeds of *C. oleifera*, and prepared in our lab with structural identification (Ye et al. 2014). 2,2-Diphenyl-1-picrylhydrazyl (DPPH) was purchased from Wako Pure Chemical Industry, Ltd (Osaka, Japan). Rotenone for inducing neurotoxicity model in animal tests was purchased from Sigma-Aldrich Company (St. Louis, MO). Superoxidase dismutase (SOD) kit, malondialdehyde (MDA) kit, acetylcholine (Ach) kit, dopamine (DA) kit and tyrosine hydroxylase (TH) kit were purchased from Nanjing Jiancheng Bioengineering Institute (Nanjing, China). Vitamin C and other experimental reagents for the analysis were of analytical purity and bought from Qianhui Chemical Company (Guangzhou, China).

### Animals

The experiments were carried out on male Kunming mice of weight 25 ± 3 g, supplied by Experimental Animal Center of South Medical University. The animals were housed under conditions of 25 ± 2 °C, 50 ± 10% humidity with a 12 h light/dark cycle. Food and water were accessible *ad libitum*. The experiments have been performed in accordance with the Chinese Guidelines for the use of laboratory animals, and received approval from the Animal Experimentation Ethic Committee of South China University of Technology. All efforts were made to minimize animal suffering and to reduce the number of animals used.

### Synthesis of iron–sapogenin

Sapogenin (1.0 g) was dissolved in 30 mL ethanol, 1 mmol sodium carbonate powder was added under 60 °C for 1 h, then mixed with 0.5 mmol FeCl_3_•6 H_2_O ethanol solution under reflux at 70 °C. The solution pH was adjusted to 8–9 to precipitate target product by ethanol/ammonia (V/V = 1). After 8 h, the precipitation was collected by centrifugation (4000 rpm, 10 min). The precipitation was washed by 1 mol/L hydrochloric acid solution and ethanol alternately and dried in vacuum.

### Structure identification

UV spectra analysis was carried out on UV-3010 ultraviolet spectrometer (Hitachi Company, Tokyo, Japan) scanning from 200 to 800 nm; IR spectra were measured on Nicolet 380 FI-IR spectrograph (Nicolet Apparatus Company, Waltham, MA) with KBr tablets from 4000 to 400 cm^−1^ with a resolution of 2 cm^−1^. Element analysis was determined on elemental analyzer Vario EL III (ELEMENTAR Company, Langenselbold, Germany).

### Morphological observation

The morphology of sapogenin metal complex was observed under a JEM-2100F high resolution scanning electron microscope (SEM) (JEOL Company, Tokyo, Japan) at 200 kV. Particle size distribution and polydispersity index (PDI) of the products were determined by Nano-2S MDT-2 Malvern Particle Size Analyzer (Malvern Instruments Ltd, Malvern, UK). Before testing, the appropriate sample was dissolved in DMSO and dispersed in water with a refractive index of 1.33. Data were calculated as the average of five repetitions.

### TG/DTG analysis

Thermogravimetric analysis (TG) was performed on Q6000-SDT Thermogravimetric Apparatus (TA Instrument Ltd, New Castle, DE). The nanoparticles (4 mg) were heated from 20 °C–800 °C under nitrogen flow at a rate of 20 °C/min. Differential thermal gravity (DTG) curve was plotted.

### DPPH radical scavenging activity

The DPPH free radical scavenging activity was analyzed by the method as reported with some modifications (Gao et al. 2008). The product was dissolved in 10 mL of 95% ethanol, and diluted to 0.025, 0.05, 0.1, 0.2, 0.4 and 0.8 mg/mL. Each sample (2 mL) was mixed with DPPH (2 × 10^−4 ^mol/L, 2 mL) in ethanol solution. The mixture was kept in the dark at room temperature for 30 min, and detected at 524 nm (absorbance *Aa*, ethanol as blank), the primary absorbance *Ao* was recorded when the extract was replaced by ethanol, and background absorbance *Ab* was recorded when ethanol took place DPPH. DPPH radical scavenging activity of the samples was calculated as follows:

DPPH radical scavenging activity (%)=[Ao-(Aa-Ab)]/Ao×100.

### Neurodegeneration induced by rotenone in mice

The experiment was performed according to the method illustrated by Murakami et al. (2014). Mice were randomly divided into six groups of eight mice for each: normal group, vehicle group, four medicated groups including sapogenin, high, middle and low doses of iron–sapogenin groups. Vehicle and medicated groups were injected subcutaneously into the back of neck with rotenone (50 mg/kg/d) for 6 weeks. Mice appeared symptoms of tremor, epilepsy, depression and other symptoms suggesting successful model. Medicated groups of mice were tail intravenously injected with the sapogenin at 100, 25 (low dose), 50 (middle dose) and 100 mg/kg (high dose) with ethanol as vehicle, respectively, for 7 days, vehicle group was injected with the same dose of ethanol instead. After behavioural test mice were sacrificed by decapitation, the half brain was quickly separated to determine the levels of MDA, SOD, Ach and DA with the ELISA method of kit description, and sapogenin content by HPLC. The other half brain was kept for immunohistochemical analysis. HPLC (Agilent Company, Santa Clara, CA) was performed in the following conditions: column: Diamonsil C18 (150 × 4.6 mm, 5 μm), mobile phase: 80% (V/V) methanol, flow rate: 0.7 mL/min, temperature: 30 °C, injection volume: 15 μL, wavelength: 208 nm.

### Behavioural test

The locomotive activities of mice were monitored by a ZZ-6 spontaneous activity apparatus (Chengdu Taimeng Software Co., China). Moving counts were collected by computer with the infrared-sensitive motion-detection system (He et al. 2009). Each mouse was placed in the testing chamber for 10 min of adaptation, followed by a 10 min recording.

### Assay of TH expression

The test was carried out on the basis of the protocol illustrated by Zhao et al. (2012). The half brain of mice was sunk in paraformaldehyde solution (pH 7.4, 40 g/L) containing 300 g/L sucrose for 48 h, and cryostat sectioned in to slices (50 μm thickness). Brain slices were incubated in TH antibody with dilution of 1:5000, stained by diaminobenzidine (DAB), and mounted to slides for photograph. Optical density of substantia nigra in the photographs was analyzed by Image-Pro Plus 6.0 software (Media Cybernetics Inc, Rockville, MD).

### Statistical analysis

Data were expressed as mean ± standard deviation, and analyzed with SPSS13.0 software (IBM, Armonk, NY). Significant tests among the groups were based on one-way analysis of variance (ANOVA) and Student–Newman–Keuls (SNK) test.

## Results

### Structure of iron–sapogenin

Sapogenin can be chelated with ferric ion in aqueous solution. The product showed single peak in HPLC with the purity of 96% on the base of peak area. It was dissolved in DMSO at 0.2 mg/mL concentration, and scanned by Ultra-violet spectrometer. Iron–sapogenin had two absorption peaks at 276 nm and 370 nm, the former is a characteristic peak of the sapogenin, and the second suggests the linkage of carboxyl group with iron leading to the appearance of a new colour group. Iron–sapogenin has stronger absorbance intensity than the sapogenin, indicating that binding and electron transfer take place between iron atom and sapogenin molecules.

**Figure 1.**
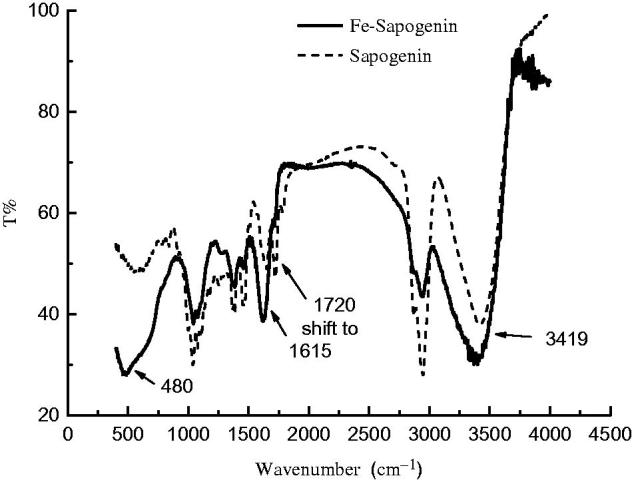
Infrared spectra of the sapogenin and iron–sapogenin.

The sapogenin showed a peak of the carbonyl group at 1720 cm^−1^ at IR spectra, but moved to 1615 cm^−1^ after chelation with iron (Figure 1). Its O–H vibration peak at 3419 cm^−1^ also became wider indicating more hydroxyl groups or water. Occurrence of a new peak at 480 nm suggested M–O vibration.

Carbon and hydrogen composition was determined with an element analyzer, and metals were measured with an atomic absorption spectrometer, the ratios of elements are listed in Table 1. It shows that four sapogenin molecules combine with one iron atom.

**Table 1.** Elemental analysis and mass percentage of metallic elements in the sapogenin and iron–sapogenin.

Compounds	Carbon (%)	Hydrogen (%)	Metal (%)	C:H:M (ratio of mol)
Sapogenin	64.49	9.979	0	30:55:0
Iron–sapogenin	64.13	8.864	2.6950	30:50:0.2702

**Figure 2.**
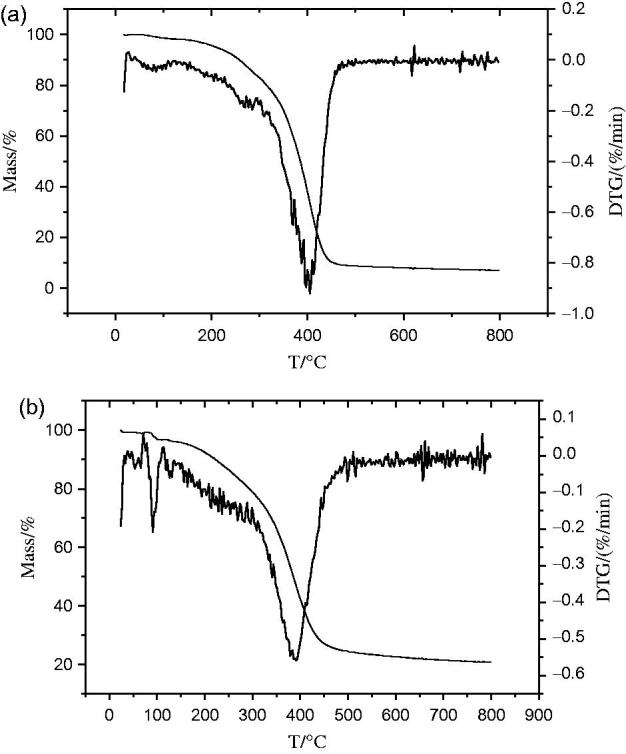
TG/DTG curves of the (a) sapogenin and (b) iron–sapogenin.

**Figure 3.**
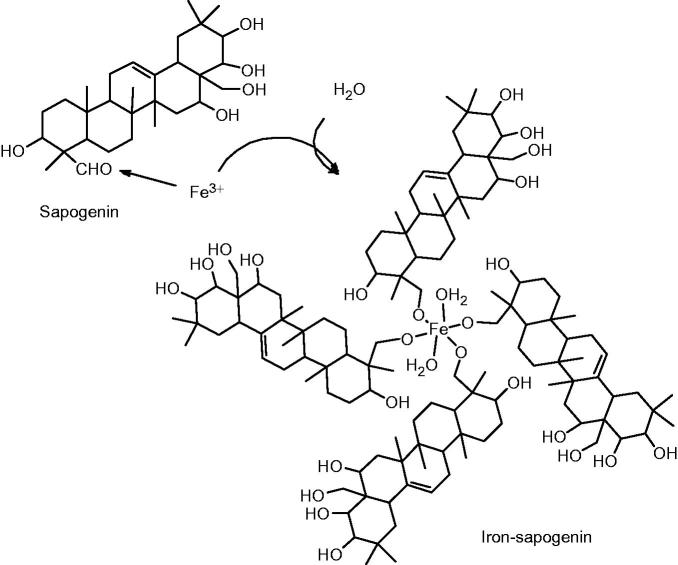
The scheme of iron–sapogenin synthesis from the sapogenin.

TG/DTG curves showed that the sapogenin lost its weight quickly from 300 °C to 430 °C without inflection point, but iron–sapogenin had inflection point at 100 °C accompanying quick weight loss from 300 °C to 470 °C and slow weight loss from 470 °C to 800 °C (Figure 2). It indicates that iron–sapogenin is more stable than the sapogenin, and two molecules of bound water in iron–sapogenin can be calculated by its weight loss. Its molecular structure can be predicted as given in Figure 3.

### Characteristics of the nanoparticles

**Figure 4.**
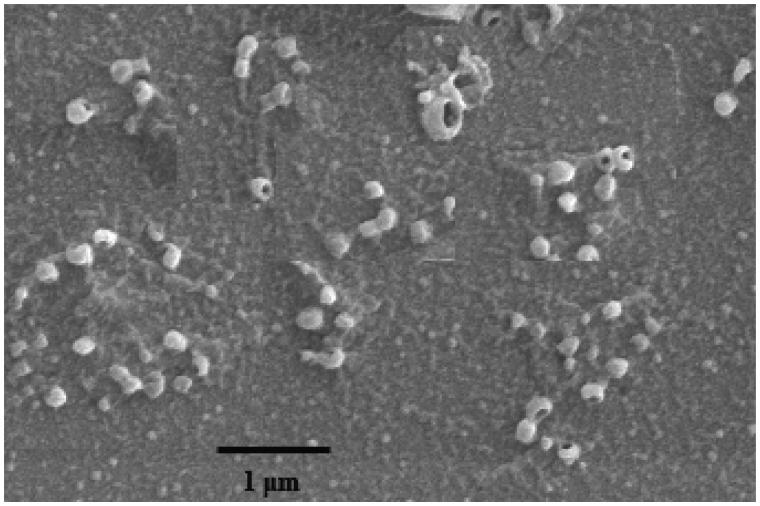
Scanning electron micrograph of iron–sapogenin at 20,000 × amplification.

Iron–sapogenin showed nanoparticle characters while dissolved in DMSO and then dispersed in water. Its size distributed in the range of 100–200 nm, and the average nanoparticle size was 162 nm. PDI of the suspension was 0.258, displaying good dispersity. Morphology of the nanoparticle was observed and photographed with SEM, and the image shows that iron–sapogenin is like a ball with a cave in it (Figure 4). The special shape indicates that four sapogenin molecules and one iron atom form a sphere with two water molecules as the cave. It means that the complex has more stable nanoparticle structure.

### DPPH radical scavenging activity of the nanoparticles

**Figure 5.**
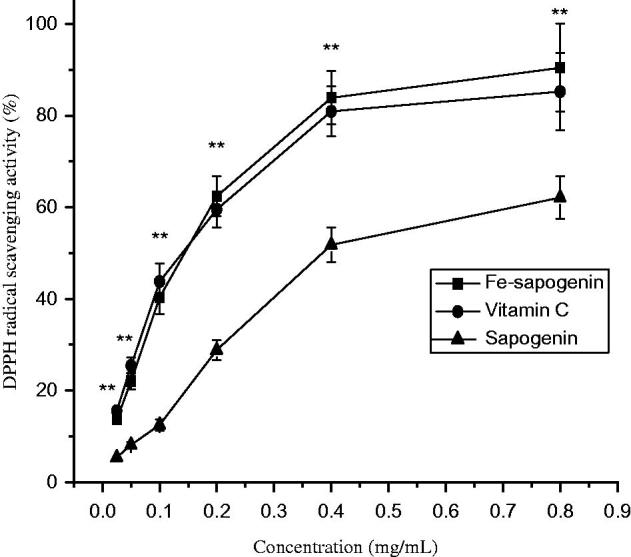
DPPH radical scavenging activity of the sapogenin and iron–sapogenin at different concentration. Data are means of three determinations ± SD. **p < 0.01, compared to the sapogenin.

DPPH determination is a classic method to evaluate free radical scavenging activity of the compounds (Musa et al. 2013). The sapogenin and its ferric complex could delete DPPH radicals at dose dependence, and iron–sapogenin had stronger activity than the sapogenin. It proves that iron strengthens electron withdrawing capacity of the sapogenin. It scavenged more than 90% DPPH radicals at 0.8 mg/mL concentration, however, vitamin C deleted about 80% at that concentration as shown in Figure 5.

### Effects of the nanoparticles on mice behaviour

Patients with neurodegeneration are usually accompanied by a lot of motor dysfunction, which can be imitated by animal tests with neurotoxicity of rotenone (Moreira et al. 2012). Rotenone is the major active ingredients and principal components of cuberesin from *Lonchocarpus utilis* used as a botanical insecticide and piscicide because of potent complex I (NADH: ubiquinone oxidoreductase) inhibitors, and known earlier to induce a PD-like syndrome after subcutaneous treatment of rats and mice (Caboni et al. 2004).

Spontaneous activities of mice, the index to assess behavioural disorder, were determined by locomotion test (Wang et al. 2014). The results showed that rotenone significantly (*p* < 0.05) lessened the spontaneous activity testifying that it could induce movement disorder. The locomotion counts were increased significantly (*p* < 0.05) after the administration of iron–sapogenin, suggesting that it alleviated the behavioural disorders induced by rotenone, and had better effects than the sapogenin on neuroprotection. The results are shown in Table 2.

**Table 2.** Effect of the sapogenin and iron–sapogenin on behaviour, antioxidant levels and neurotransmitters in brain of mice.

Group	Dosage(mg/kg)	MDA (nmol/mg of protein)	SOD (U/mg of protein)	Locomotivecount	Opticaldensity	Ach (μmol/mg of protein)	DA (nmol/mg of protein)	Sapogenin(ng/g)
Normal control	/	7.55 ± 1.39^b^^,c^	121.20 ± 17.15^b^^,c^	147.4 ± 21.7^b^^,c^	0.114 ± 0.007^b^^,c^	9.04 ± 0.59^b^^,c^	82.65 ± 5.64^b^^,c^	/
Vehicle	/	13.11 ± 0.79^a^^,c^	90.21 ± 7.84^a^	67.4 ± 15.7^a^	0.038 ± 0.009^a^	5.81 ± 0.28^a^^,c^	61.25 ± 3.53^a^	/
Sapogenin	100	10.78 ± 1.06^a^^,b^	97.85 ± 7.24^a^^,b^	86.2 ± 16.5^a^^,b^	0.057 ± 0.008^a^	7.53 ± 0.41^a^^,b^	66.54 ± 3.76^b^	2.31 ± 0.57
Iron-sapogenin	25	9.96 ± 1.73^a^^,b^	103.53 ± 9.26^a^^,b^	89.6 ± 13.8^a^^,b^	0.066 ± 0.005^a^^,b^	7.85 ± 0.36^a^^,b^	67.22 ± 3.94^a^	2.15 ± 0.64
Iron-sapogenin	50	9.84 ± 1.46^a^^,b,c^	107.82 ± 9.13^a^^,b,c^	93.5 ± 14.3^a^^,b^	0.072 ± 0.007^a^^,b,c^	8.04 ± 0.42^b^	71.53 ± 3.74^a^^,b^	5.26 ± 1.15^c^
Iron-sapogenin	100	9.81 ± 1.17^a^^,b,c^	110.17 ± 10.21^b^^,c^	108.4 ± 16.7^a^^,b,c^	0.078 ± 0.009^a^^,b,c^	8.26 ± 0.47^b^^,c^	75.27 ± 4.54^b^^,c^	11.42 ± 2.32^c^

MDA: malondialdehyde; SOD: superoxidase dismutase, Ach: acetylcholine; DA: dopamine. Data are presented as mean ± standard deviation (*n* = 8).

a *p* < 0.05 as compared to normal group.

b *p* < 0.05 as compared to veh group.

c *p* < 0.05 as compared to sapogenin group.

### Antioxidant level in brain tissues of mice

MDA and SOD are always used to deduce free radical or antioxidant levels *in vivo* (Zhou et al. 2014). Mice with rotenone had higher level of MDA and lower SOD activity in brain, indicating that rotenone increased free radicals *in vivo*. A significant (*p* < 0.05) reduction of MDA content and increase of SOD activity in brain were observed in groups of iron–sapogenin compared with the vehicle (Table 2), revealing that the complex of sapogenin with iron is more effective in eliminating free radicals *in vivo* than the sapogenin.

### Neurotransmitters in brain tissues of mice

Dopamine (DA) and acetylcholine (Ach) are important neurotransmitters related to PD, AD and other neurodegeneration (Calabresi et al. 2000). Rotenone caused decrease of DA and Ach level in brain, and led to injury of neurons. Sapogenin and iron–sapogenin both prevented the reduction of DA and Ach induced by rotenone, especially iron–sapogenin increased DA and Ach levels in dose dependence, and had no significant difference with normal mice at the dose of 100 mg/kg, showed the stronger neuroprotective effect (Table 2).

### Protection of TH by the nanoparticles

**Figure 6.**
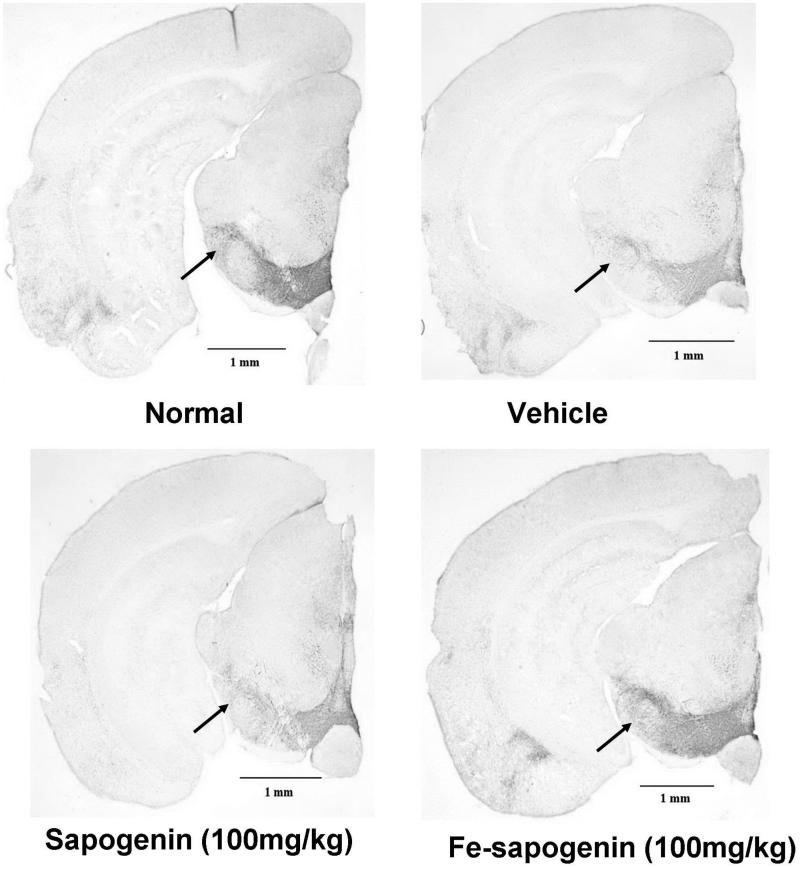
Tyrosine hydroxylase (TH) immunohistochemistry of substantia nigra sections in mice with different treatments. The photos were taken by camera in 50× magnification.

Tyrosine hydroxylase (TH) is the key enzyme for the synthesis of monoamines neurotransmitters such as dopamine and noradrenaline in neurons, and its expression is lowered in brain tissues of patients with neurodegenerative diseases (Kastner et al. 1993). The expression of TH can be reflected by immunostaining intensity of brain slices in immunohistochemistry. Rotenone-treated mice had less staining in the substantia nigra of brain slices as compared with normal mice, indicating that rotenone depressed the expression of TH (Figure 6). The optical density of the substantia nigra is listed in Table 2. The optical intensity has increased from low dose to high dose of the iron–sapogenin. It proves that iron–sapogenin exerts its protective effect on neurons.

## Discussion

The neuroprotection of iron–sapogenin is attributed to free radical elimination *in vivo*. Free radicals with one or more unpaired outer shell electrons are extremely reactive and generally highly unstable. Reactive oxygen species, such as superoxide radical, hydrogen peroxide, hydroxyl radical, and singlet oxygen are main free radicals in body and of the greatest biological significance (Gutowski & Kowalczyk 2013). They are extremely reactive and potentially damaging transient chemical species. Free radicals generated by mitochondrial metabolism can be removed by redox system *in vivo* in healthful condition, however, the balance is broken in illness, and excess free radicals keep active, injure normal brain tissues and induce neurodegeneration (Labunskyy & Gladyshev 2013). Some antioxidants show free radical scavenging activities *in vitro*, but have little activity *in vivo*, which is crucial to therapeutic effects (Kasote et al. 2015). In this experiment, significant changes of MDA and SOD suggest that iron–sapogenin takes effect on neuroprotection through elimination of free radicals in brain.

On view of molecular structure, iron–sapogenin has compact organometallic structure with fewer metals in the centre, and strengthens electron transmittance between iron atom and the sapogenin molecules, which enhances free radical scavenging activity. Inorganic metals and metabolic disorders such as defective lipid metabolism may lead to metal accumulation and neurotoxicity (Nday et al. 2012; Colombelli et al. 2015), but iron–sapogenin are organic metallic compounds and beneficial to neuroprotection.

Sapogenin content in mice brain increases in dose dependence of iron–sapogenin (Table 2), and is about five times in iron–sapogenin group more than sapogenin group at the same dose (100 mg/kg). Iron may increase the permeability of blood–brain barrier through the upregulation of transferrin receptor mRNA after treatment with the iron chelator (Hersom et al. 2016). It is the possible mechanism of greater neuroprotective effects caused by iron–sapogenin complex.

## Conclusion

The sapogenin, a bioactive compound isolated from the seeds of *C. oleifera*, can react with iron to form the chelate of iron–sapogenin. Its structure is deduced as four sapogenin molecules binding to one iron atom with two water molecules. It possesses nanoparticle characters with spherical shape. The linkage of sapogenin with iron strengthens electron transfer among molecules, improves free radical scavenging activity. Iron–sapogenin protects brain from damage caused by neurotoxicity of rotenone, plays an important role in antioxidative level in central tissues. It is a good candidate as a drug for neuroprotection.
